# Saving Limited Resources During Covid-19 Pandemic

**DOI:** 10.37825/2239-9747.1003

**Published:** 2020-10-01

**Authors:** O Piazza

**Affiliations:** Department of Medicine, Surgery, Dentistry, University of Salerno, Italy

**Keywords:** COVID-19, Ethics, ICU

## EDITORIAL

“Age (*is*) an important factor in making the terrible choice of who will receive scarce resources in a pandemic.”, wrote Professor Arthur Caplan, Director of the section of Medical Ethics at the New York University-Grossman School of Medicine [1]. This opinion, if extrapolated from its context, would be immediately rejected as inhuman and unacceptable by anyone, medical or lay people, young or old. However, in Italy, the SARS-CoV-2 pandemic was marked by the severe lack of personal protective equipment (PPE), mechanical ventilators, hospital beds and in particular ICU beds, and this resulted in an inevitable selection of patients. ICU physicians, often by themselves, face this situation, when ER request exceeds the availability of beds and mechanical ventilators in the area, also before the pandemic. This problem has been aggravated by COVID-19, and it is now known and feared by the large audience. If maximizing the number of saved lives is the common societal objective, and when epidemiological and clinical data support the risk of failure, can age lawfully be used for the allocation of a valuable resource as a mechanic ventilator? Simplifying, if there is an equal need between two patients, age can be the decisive element in defining the priority of treatment: lifesaving procedures, such as intubating and ventilating, will be carried out only in younger patients, reserving only less invasive or palliative treatments for the elderly. Following this principle, the elderly, lesser valued citizens, would give young people the right to play their game of life, as defined by the principle of “fair innings”, or fair life expectancy. Is the age of patients the right choice when it is selected as a triage criterion?

In my opinion, age must never be the main factor that determines a person’s right to intensive care, since it is an unreliable and insufficient index of the patient’s ability to respond to intensive care and to recover autonomy functional. A healthy 75-year-old cannot be denied access to resuscitation treatment on the basis of age alone, although elderly patients with severe respiratory insufficiency secondary to COVID-19 have a high probability of dying despite intensive care and, consequently, they may have a lower priority for admission to intensive care in conditions of irremediable and extreme shortage of beds. The Italian Society of Anesthesia (SIAARTI) has published a document entitled “clinical ethics recommendations for the breakdown of intensive care treatments, in exceptional circumstances limited to resources” in partial agreement with Professor Caplan. In this document, the principle of “*saving limited resources, which can become extremely scarce, for those who have a much greater chance of survival and life expectancy, in order to maximize the benefits for the greatest number of people” is stated. COVID 19 acute respiratory disease in frail elderly patients has a long course, and outcomes are more malignant than in healthy young subjects. SIAARTI, therefore, suggested that: “together with the age, comorbidity and functional status of each patient in critical conditions must be carefully evaluated in these exceptional circumstances*”.

The British guidelines of the National Institute of Health and Care Excellence (NICE), updated to the 29^th^ of April 2020, suggest reserving intensive care only for patients over 65 with a low fragility score, while considering very selectively hospitalization in ICU for over sixty-five frail patients. A score greater than 5 on the Clinical Frailty Scale (CFS) should discourage attempting invasive approaches or “wasting” a mechanical ventilator for a patient who needs assistance for climbing stairs, washing or dressing. In this pandemic, the ethical obligation to prioritize the well-being of individual patients could be surmounted by public health policies that push to do the greater good for the largest number of patients. White and Lo [2] support the approach of giving priority to critically ill patients who are more likely to survive at discharge too. Defining a rigid cut-off - a precise threshold of age and CFS score - are, in my opinion, more “defensive” tools for young and inexperienced doctors, left in distress in the emergency room devastated by the epidemic, rather than elements of ethics to reflect on.

Again, I repeat that it is essential that these decisions are based on clinical factors related to therapeutic outcomes and not on the basis of discriminatory judgments about the value of individual lives. Likewise, a simplistic age-based or disability-based withdrawal system would not only be unethical, but also illegal, since it would constitute a discrimination. These decisions are extremely distressing for both those affected and those forced to make them. Professor Aldo Masullo, a great philosopher, who died a few days ago at the age of 97, wrote about the COVID epidemic: “*the shortage of life, the shortage of time, are the exceptional conditions in which we find ourselves nailed by this external affair, and all the more, I repeat, this feeling is strong, as we live in an era of advanced technologies. How is it possible that, nowadays, we must succumb to what we do not know? We have the means to go to the moon, to Mars, to make great interplanetary journeys, and yet we surrender in front of a tiny living being, as this virus of which we are now prisoners. ... Later, the big topic of the economic disaster will follow, but this is outside the scope of our conversation*” (Interview by Fiorinda Li Vigni19 March 2020).

An epidemic is not only a disease but a social crisis, it is not a mere problem of ICU beds, but a humanitarian emergency. We need a long-term plan to safely treat COVID-19 and non-COVID patients, firstly as outpatients, at their home, and in hospitals, whenever it is necessary. Having tackled the first pandemic wave, we now have the tools to plan, and organize, and rationalize resources, such that the next hyper-inflow of COVID patients to the hospitals or a possible, future pandemic linked to the next small, tiny, virus will not find us dismayed and defenceless.
